# Use of TLC and Computational Methods to Determine Lipophilicity Parameters of Selected Neuroleptics: Comparison of Experimental and Theoretical Studies

**DOI:** 10.3390/ph18091255

**Published:** 2025-08-24

**Authors:** Daria Klimoszek, Małgorzata Dołowy, Małgorzata Jeleń, Katarzyna Bober-Majnusz

**Affiliations:** 1 Department of Analytical Chemistry, Faculty of Pharmaceutical Sciences in Sosnowiec, Medical University of Silesia in Katowice, Jagiellońska Street 4, 41-200 Sosnowiec, Poland; bober@sum.edu.pl; 2 Department of Organic Chemistry, Faculty of Pharmaceutical Sciences in Sosnowiec, Medical University of Silesia in Katowice, Jagiellońska Street 4, 41-200 Sosnowiec, Poland; manowak@sum.edu.pl

**Keywords:** lipophilicity, neuroleptics, nitrogen containing compounds, thin-layer chromatography, topological indices

## Abstract

**Background:** Compound lipophilicity is a fundamental physicochemical property for determining the pharmacokinetic and pharmacodynamic profiles of therapeutic substances. It is successfully used in the early stages of drug candidates’ design and development. **Aim**: Taking into account the importance of this parameter, we aimed to assess and compare the utility of a hybrid procedure based on calculation methods and an experimental one for rapid and simple estimation of the lipophilicity of selected neuroleptics such as fluphenazine, triflupromazine, trifluoperazine, flupentixol and zuclopenthixol and their potential new derivatives. **Methods:** Log P values of the studied compounds were predicted by means of different platforms and algorithms: AlogPs, ilogP, XlogP3, WlogP, MlogP, milogP, logPsilicos-it, logP_consensus_, logP_chemaxon_ and logP_ACD/Labs._ The experimental determination of lipophilicity was carried out by reverse-phase thin-layer chromatography (RP-TLC) using three types of stationary phases—RP-2F_254_, RP-8F_254_ and RP-18F_254_—and mobile phases consisted of acetone, acetonitrile and 1,4-dioxane as organic modifiers. **Results:** Our results provide a confident proposal of optimal chromatographic conditions to experimentally determine the lipophilicity of neuroleptic drugs, including new derivatives. **Conclusions:** Additionally, for the first time, the paper shows the application of selected topological indices in determining lipophilicity factors and other ADMET parameters of neuroleptics and, in the future, the newly synthesized quinoline derivatives of the studied compounds.

## 1. Introduction

Neuroleptics are a chemically diverse group of heterocyclic compounds containing a nitrogen atom. Classical neuroleptics consist of phenothiazine, thioxanthene, dibenzoxepine, dihydroindole or a benzamide structure [1] of phenothiazine and thioxanthene derivatives. They influence the central nervous system. Some drugs such as fluphenazine, triflupromazine, flupentixol and zuclopenthixol can be distinguished among them. Fluphenazine is a psychotropic drug that belongs to the group of first-generation typical antipsychotic agents [2]. It is a phenothiazine derivative that has a trifluoromethyl group connected to one of the benzene and piperazine rings with a 2-hydroxyethyl group as a substituent. It is present in pharmaceutical preparations in the form of fluphenazine hexanoate and fluphenazine decanoate. It blocks dopamine receptors, specifically D_1_ and D_2_ receptors in the brain, and is used in the treatment of psychoses, schizophrenia and bipolar disorder and in long-term neuroleptic therapy [1]. Fluphenazine, as a drug, can be administered in the form of pills or intramuscular injections. The intramuscular injection can contain short-acting or long-acting forms of fluphenazine [1,2,3,4,5]. Triflupromazine is an antipsychotic drug. It is the phenothiazine derivative that has the trifluoromethyl group connected to one of the benzene rings and a tertiary amine as the substituent. It also blocks dopamine receptors in the brain. It binds 5-hydroxytryptamine receptor 2B (5-HT_2B_), muscarinic acetylcholine receptor M_1_ and muscarinic acetylcholine receptor M_2_. It is used to treat psychoses by controlling aggressive behavior that occurs during these episodes [1,6]. Additionally, they may also be applied to control vomiting, nausea and severe hiccups. Triflupromazine hydrochloride as a drug can be administered in the form of pills or injections [1,6,7]. The next substance, trifluoperazine dihydrochloride, is a salt of another phenothiazine derivative. It is used to treat schizophrenia and anxiety in the form of tablets or an injectable solution.

**Figure 1:**
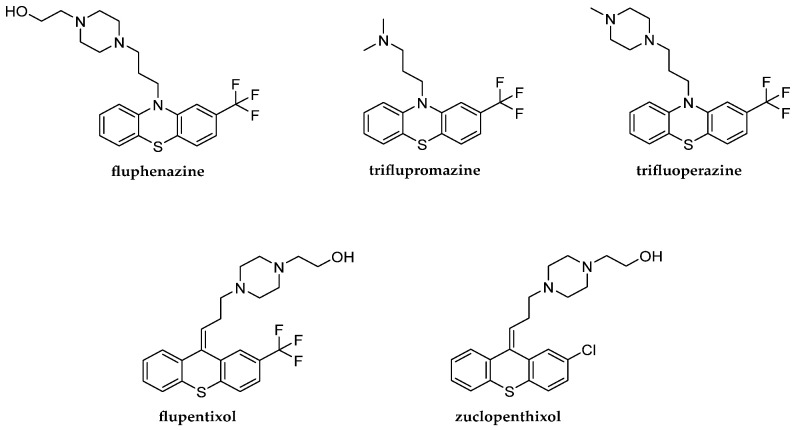
Structural formulas of fluphenazine, triflupromazine, trifluoperazine, flupentixol and zuclopenthixol.

Flupentixol is a psychotropic drug that belongs to the group of the first-generation typical antipsychotics [1,8]. It is a structural analog of fluphenazine. It is also a thioxanthene derivative that has a trifluoromethyl group connected to one of the benzene and piperazine rings with a 2-hydroxyethyl group as a substituent. Flupentixol occurs as a *trans*-isomer and a *cis*-isomer, but only the *cis*-isomer is used as a component of drugs due to its pharmacological activity. It is mainly produced as flupentixol dihydrochloride and flupentixol decanoate. It blocks various dopamine receptors. It also blocks histamine, adrenaline and serotonin receptors. It is applied to treat schizophrenia, psychoses, apathy and neurosis and also as an antidepressant. Flupentixol as a drug can be administered in the form of pills or a long-acting intramuscular injection [1,7,8]. Zuclopenthixol is a first-generation typical antipsychotic [1,9]. It is a thioxanthene derivative that has a chlorine connected to one of the benzene and piperazine rings with a 2-hydroxyethyl group as a substituent. It is the *cis*-isomer of clopenthixol. It is present in pharmaceutical formulations such as zuclopenthixol dihydrochloride, zuclopenthixol acetate and zuclopenthixol decanoate [1,9]. It blocks dopamine receptors in the brain and also blocks histamine, adrenaline and serotonin receptors. It acts against psychoses and schizophrenia. It has antianxiety activity. Zuclopenthixol as the drug can be administered in the form of pills or a long-acting intramuscular injection [1,10,11,12]. The chemical structures of the tested compounds are shown in Figure 1.

Currently available neuroleptics have certain limitations. The long-term use of neuroleptics, including those presented above, may cause many adverse effects such as gynecomastia, involuntary movement, metabolic, weight gain and impotence [1]. Therefore, rapid and efficient methods for analyzing parameters important for determining the ADMET profile (i.e., absorption, distribution, metabolism, elimination and toxicity) of known neuroleptic drugs or new candidates for antipsychotic medications are meaningful. Recent research shows that lipophilicity is a significant factor in determining the bioavailability, pharmacokinetics and toxicological profile of molecules. It is one of the key properties to address in drug design and development [13,14].

Some studies have described a strong correlation between lipophilicity parameters and the bioactivity, pharmacokinetics and pharmacodynamic properties of drug substances [15,16,17,18,19,20,21,22,23]. Generally, there are two methods dedicated to estimating the lipophilicity parameter (logP), i.e., calculation “in silico” and experimental methods. Among the experimental procedures, reverse-phase thin-layer chromatography (RP-TLC) is the simplest chromatographic technique possible to use for the determination of the experimental value of the lipophilicity factor of organic molecules, including antipsychotic agents. The chromatographic parameter R_MW_ can be interpreted as a logP value. A non-polar stationary phase, such as RP-18 or the less hydrophobic RP-8, and various organic modifiers like n-octanol, 1,4-dioxane, acetonitrile, methanol, acetone and tetrahydrofuran as mobile phase components are used for lipophilicity measurements.

**Figure 2:**
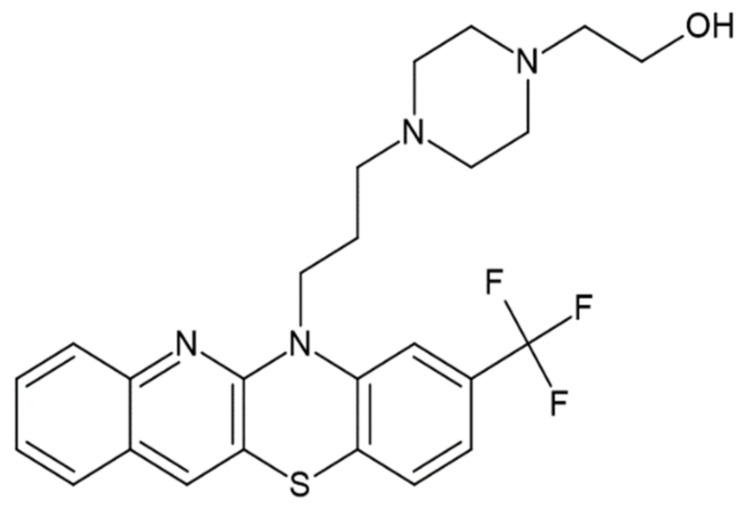
The structure of the new derivative.

The aim of the work was to assess and compare the lipophilicity parameters of five neuroleptics—fluphenazine, triflupromazine, trifluoperazine, flupentixol and zuclopenthixol—obtained by means of both computational and chromatographic methods. The lipophilicity parameter (R_MW_) values were determined using various chromatographic systems for RP-TLC and compared with theoretical logP values predicted using computational algorithms (AlogPs, ilogP, XlogP3, WlogP, MlogP, milogP, logPsilicos-it, logPconsensus, logP_chemaxon_ and logP_ACD/Labs)_ [24,25,26,27]. Furthermore, this is the first time that selected topological indices for the studied molecules were calculated without using ready-made programs. The newly calculated topological indices for these compounds based on the distance matrix and the adjacency matrix, respectively, such as Pyka (*A*, ^0^*B*, ^1^*B*), Wiener (*W*), Rouvray–Crafford (*R*), Gutman (*M*, *M*^ν^) and Randić (^0^*χ*, ^1^*χ*, ^0^*χ*^ν^, ^1^*χ*^ν^), were discussed in terms of their correlation with lipophilicity factors and other ADMET factors. The usefulness of the hybrid method, i.e., experiments and calculations for predicting key parameters for ADMET, including lipophilicity, was evaluated and discussed for the five tested compounds known as active substances and for designing their new derivatives containing, for example, a quinoline structure, as shown in Figure 2. 

## 2. Results and Discussion

During the first step of our study, we tried to use in silico methods available in the form of both ChemSketch and Molinspiration Cheminformatics software to predict selected physicochemical parameters of the studied compounds [26,27]. As shown in Figure 1, the studied antipsychotic agents belong to known phenothiazine and thioxanthene derivatives, respectively. The structure of new proposed derivatives of these is shown in Figure 2. An analysis of the selected physicochemical parameters of these substances, listed in Table 1, indicates a similarity between the compounds under study except trifluoropromazine, which is characterized by lower values of all these parameters. This is probably due to a different structure, i.e., the presence of aliphatic substituents, compared to the other compounds studied. The results presented in Table 1 confirm the suitability of applied in silico tools for rapid estimation of the main physicochemical properties of bioactive compounds containing a phenothiazine and thioxanthene structure, as well as the newly designed structure shown in Figure 2, additionally containing a quinoline ring, as a potential new drug candidate. The highest similarity in the calculated parameters is observed for thioxanthene derivatives, i.e., flupentixol and zuclopenthixol. However, some differences, particularly about not only the molar mass values but also the TPSA parameter from 8.17 to 11.41, are visible for the members of the second group, i.e., phenothiazine derivatives such as fluphenazine, triflupromazine and trifluoperazine. TPSA is a strong factor in the study of drug transport properties such as intestinal absorption. The results, including the Topological Polar Surface Area of the new proposed molecule, show that the new derivative with a TPSA lower than 50 Å^2^ is associated with better blood–brain barrier penetration, allowing it to reach the central nervous system (CNS). Furthermore, it is worth noting that the newly proposed molecule has a molecular weight of less than 500 Da, which also preliminarily confirms its potential oral bioavailability as a promising drug candidate. Considering the impact of lipophilicity on the ADMET characteristics of drugs and the lack of accurate data in the literature on the method of determining the experimental value of this parameter for selected of studied compounds and especially zuclopentixol, we used theoretical methods to determine the lipophilicity parameter as a decimal logarithm of the partition coefficient (logP) for zuclopentixol and other studied compounds i.e., fluphenazine, triflupromazine, trifluoperazine and flupentixol. In addition to this, the partition coefficient values were predicted for the new structure. The following in silico tools, such as DrugBank, SwissADME, ChemSketch and Molinspiration Cheminformatics, were successfully applied to determine the partition coefficient in the form of logP as AlogPs, ilogP, XlogP3, WlogP, MlogP, milogP, logPsilicos-it, logP_consensus_, logP_chemaxon_ and logP_ACD/Labs_ [24,25,26,27]. All obtained partition coefficients and their average values (logP_avg)_ are summarized in Table 2.

**Figure 3:**
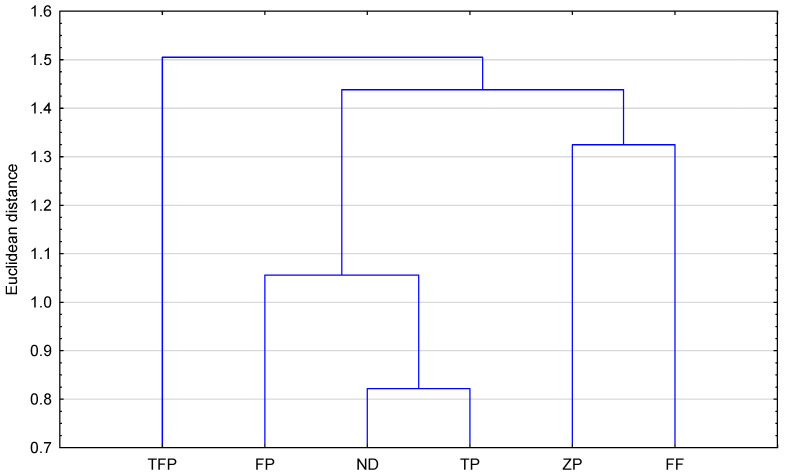
Similarity analysis of examined compounds including the newly proposed structure on the basis of calculated logP values (ilogP, XlogP3, WlogP, MlogP, milogP, logPsilicos-it, logP_consensus_, logP_ACD/Labs_) (FF—fluphenazine, TFP—triflupromazine, TP—trifluperazine, FP—flupentixol, ZP—zuclopenthixol, ND—new derivative).

Figure 3 shows that the theoretically obtained partition coefficients (ilogP, XlogP3, WlogP, MlogP, logPsilicos-it, logPconsensus, logP_ACD/labs,_ milogP and their average values) allow grouping of the examined compounds into two visible clusters. The greatest similarity is between ND and TP, as well as FP. They form the first cluster in Figure 3. The weakest similarity is observed with TFP. The second cluster is formed by ZP and FF. This confirms the early observations based on comparing of all logP_avg_ given in Table 2. Further analysis of the obtained logP values allowed for designing a correlation matrix between all these results. Table S1 (Supplementary Materials) presents the results of the correlation matrix prepared. As can be observed based on all calculated logP values, the best linear correlation (*p* < 0.05) shows the following: MlogP and logP_Chemaxon_ (R = 0.9651), MlogP and logP_Consensus_ (R=0.9844), AlogPs and milogP (R = 0.9642), milogP and logP_Chemaxon_ (R = 0.9931). The average logP value (logP_avg_.) shows good correlation with logP_Chemaxon_ (R = 0.9741), with WlogP (R = 0.9566) and with logP_Consensus_ (R = 0.9524), as well as with milogP (R=0.9731). Of all calculated logP values, the highest correlation with logP_exp_ (logP in n-octanol-water) is indicated for AlogPs (R = 0.9552) and XlogP3 (R=0.9984). Therefore, these correlations can be helpful in the future to determine the unknown logP_exp_ for ND and ZP.

Table 2 shows that among the ten calculation algorithms, the biggest similarity to logP_exp_ available in DrugBank for fluphenazine, flupentixol, trifluoperazine and flupromazine indicates XLOGP3. This fact confirms the highest predictive power of the proposed method. The lowest values of all are seen for ilogP (3.52 for triflupromazine to 4.10 for zuclopentixol and 4.41 for new derivative). It resulted in the most significant differences compared to logP_exp_ for these compounds. A difference between the logP values, from 3.52 (ilogP) to 6.03 (WlogP), can be noted in the case of triflupromazine. The highest differences with logP_exp_ generate the data obtained in the form of ilogP, MlogP and logP_silicos-it_. This fact confirms the necessity of comparing the calculated parameters with the parameters obtained from experiments. Analysis of the average values of logP, i.e., logP_avg_, calculated on the basis of partition coefficients predicted in silico using various calculation algorithms indicates that the lipophilicity of the studied neuroleptic agents increases in order: fluphenazine < zuclopentixol < flupentixol < trifluoperazine < tiflupromazine. The logP_avg._ value obtained for the new derivative indicates the biggest similarity to flupentixol. The results of logP_avg_ of fluphenazine, flupentixol trifluoperazine and tiflupromazine are similar to those of logP_exp_ (logP in n-octanol-water) available in DrugBank for these four of the five studied compounds [24]. This situation confirms that the applied calculation algorithms can be useful in rapid estimation of the lipophilic properties of tested molecules and related compounds. The obtained average value of logP for zuclopentixol shows that this compound possesses relatively lower lipophilicity, similar to fluphenazine. Similarity analysis of all studied compounds based on the selected logP calculated by using available algorithms such as ilogP, XlogP3, WlogP, MlogP, milogP, logPsilicos-it, logP_consensus_, logP_ACD/Labs_ and logP_avg_ is presented as a dendrogram in Figure 3.

When considering a substance as a drug or drug candidate, respectively, it is also essential to determine the blood–brain barrier permeability parameter (logBB) as well as logPS factor, which describes the penetration of the drug into the CNS (central nervous system). Therefore, in the next stage of our research, we proposed an in silico approach—the Percepta platform—to predict both parameters for the studied neuroleptic agents and the new derivative presented in Figure 2. The results listed in Table 3 show that for all tested molecules, logBB is in the range of 0.106 (new derivative) to 0.847 (trifluoperazine), and logPS > −2 (−1.940 ÷ −1.190). It means that not only the tested drugs but also newly proposed structures will penetrate the nervous system. The other parameters predicted by using the Percepta platform were the human skin permeability coefficient (logKp) and the Caco-2 cell permeability factor (Papp value, human colon adenoma cells). The results of logKp in Table 3, ranging from −2.557 (for triflupromazine) to −2.755 (for zuclopentixol), confirm the relatively low skin permeability of all studied molecules, including the new one. However, the Papp values ranging from 53 × 10^−6^ to 221 × 10^−6^ cm/s indicate the good intestine permeability of the five examined drugs and their new derivative.

As is known, some neuroleptics can act as inhibitors of the P450 (CYP) enzyme family, especially CYP3A4 (found in the liver and the intestine) and CYP2D6 (which metabolizes about 25% of medications, including antidepressants and antipsychotics) and can affect their interaction with other drugs; thus, it is essential to develop a cheap, fast and accurate method including in silico models to assess the possible interaction of neuroleptics with cytochromes P450. This study evaluated the usefulness of in silico methods for determining the action of the compounds studied, including the newly proposed structure with the following cytochrome P450, such as CYP1A2, CYP2C9, CYP2C19, CYP2D6 and CYP3A4—as shown in Table 3. The data listed in Table 3 confirm that the Percepta platform is a cheap and rapid tool to estimate the interaction of all tested molecules, including the new one, with CYP450. All of the examined substances are CYP3A4 inhibitors. Almost all of them can interact with other enzymes, i.e., CYP1A2, CYP2C9, CYP2C19 and CYP2D6.

In the final stage of in silico research, various topological indices were calculated for the compounds under study (Table 4). The values of these indices vary depending on the method used. As shown in Table 4, the index R-Rouvray–Crafford has the highest value, ranging from 1984.308 to 5603.378, and the index ^1^B has the lowest (0.2492–0.3498). The Randić indices (^0^*χ*, ^1^*χ*, ^0^*χ*^ν^, ^1^*χ*^ν^) are the closest to each other.

**Figure 4:**
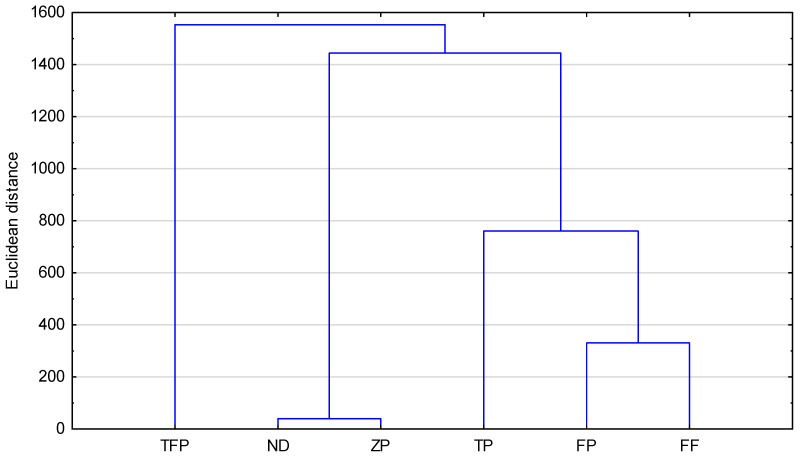
Similarity analysis of examined compounds on the basis of calculated topological indices (FF—fluphenazine, TFP—triflupromazine, TP—trifluoperazine, FP—flupentixol, ZP—zuclopenthixol, ND—new derivative).

Figure 4 presents the results of the similarity analysis (Euclidean distance) of the studied compounds based on the calculated topological indices. As can be observed, all compounds studied, including the newly proposed structure, form two groups. To the first one belong TFP—triflupromazine; ZP—zuclopenthixol; and ND—new derivative. The second group is formed by TP—trifluoperazine; FP—flupentixol; and FF—fluphenazine. The greatest similarity (the smallest Euclidean distance) is shown by ZP and ND (zuclopenthixol and new derivative). This fact confirms that topological indices can be useful to group the selected neuroleptics.

As part of the continuation of this study, we attempted to use the experimental, i.e., chromatographic method to determine the lipophilicity parameter of five commercially available neuroleptics: fluphenazine, triflupromazine, trifluoperazine, flupentixol and zuclopenthixol. As described in the Materials and Methods Section, we successfully applied thin-layer chromatography in a reverse-phase system (RP-TLC) and various systems containing chromatographic plates: RP-2F_254_, RP-8F_254_ and RP-18F_254_. As mobile phases, mixtures of acetone, acetonitrile or 1,4-dioxane and TRIS buffer (pH=7.4) were used. These chromatographic conditions consisted of silica gel based stationary phases modified with aliphatic hydrocarbons (C2, C8, C18) and mixtures of organic solvents with the buffer TRIS at physiological pH (7.4) which were the most popular and effective in lipophilicity studies conducted by various analysts and have also been successfully used in our previous work [15,16,17,18,19,20,23]. The chromatographic parameter of lipophilicity R_MW_ obtained under these conditions, using Soczewiński–Wachtmeister’s equation, is presented in Table 5. The data for the linear equation between the content of the organic modifier and R_M_ values obtained by using Soczewiński–Wachtmeister’s equation (R_M_ = R_MW_ + S∙φ) are presented in the Supplementary Materials as Tables S2–S6. Sufficient linearity of these relationships was confirmed by a high correlation coefficient (r), significant p-values, a low standard error of estimate and a high Fisher F-distribution value.

**Figure 5:**
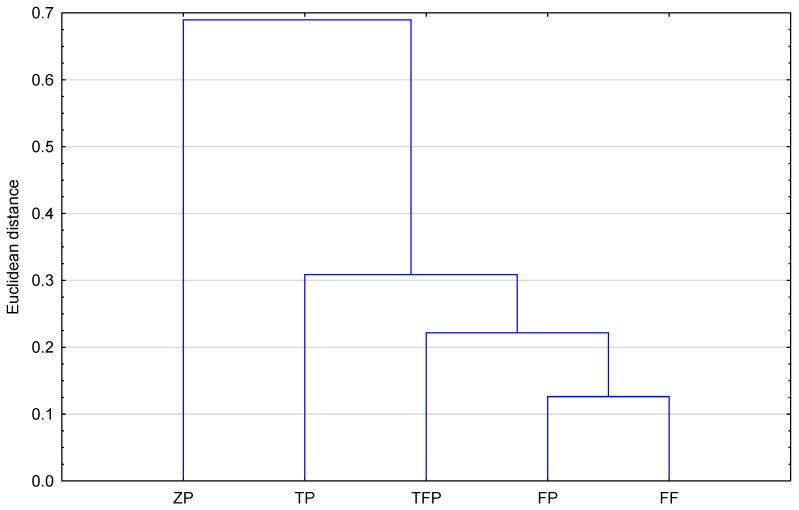
Dendrogram of similarity of the tested compounds based on their R_MW_ obtained by using acetone–TRIS buffer mobile phase (FF—fluphenazine, TFP—triflupromazine, TP—trifluperazine, FP—flupentixol, ZP—zuclopenthixol).

Interestingly, the chromatographic parameters of lipophilicity listed in Table 5 were found to be lower compared to those predicted by the use of calculation methods, such as logP values. A cluster analysis was used to compare the similarity between the investigated compounds. An exemplary dendrogram of all compounds tested, prepared based on R_MW_ values by using acetone–TRIS buffer as mobile phase, is presented in Figure 5. It confirms the biggest similarity of FF (fluphenazine) and FP (flupentixol). They form a cluster with the smallest Euclidean distance. The most distant (least similar) to them in terms of lipophilicity on the dendrogram is ZP (zuclopenthixol). Comparing exactly the chemical structures of the analyzed compounds and their chromatographic lipophilicity parameter (R_MW_), a certain relationship can be observed. Flupentixol and zuclopenthixol differ in the type of substituent in the benzene ring. Flupentixol has trifluoromethyl as the substituent whereas zuclopenthixol has chlorine as the substituent. That difference causes flupentixol’s chromatographic parameters of lipophilicity to be higher than zuclopenthixol’s chromatographic parameters of lipophilicity determined by the use of RP-TLC on the TLC plates RP-2F_254_, RP-8F_254_ and RP-18F_254_ as the stationary phase and acetone–TRIS buffer, acetonitrile–TRIS buffer and 1,4-dioxane–TRIS buffer as the mobile phase. The only exception is zuclopenthixol’s chromatographic parameter of lipophilicity determined by the use of RP-TLC on the TLC plate RP-18F_254_ as the stationary phase and acetonitrile–TRIS buffer as the mobile phase which is higher than flupentixol’s chromatographic parameter of lipophilicity obtained in the same conditions. Another dependence can be observed between trifluoperazine and fluphenazine. Fluphenazine has an additional methylene group and hydroxyl group to trifluoperazine which results in lower values of chromatographic parameters of lipophilicity determined by the use of RP-TLC on each TLC plate and mobile phase. Other dependence can be pointed out between flupentixol and fluphenazine. Fluphenazine has nitrogen across from sulfur in its structure and it does not have double bond in that area in comparison to flupentixol. The majority of fluphenazine’s chromatographic parameters of lipophilicity are lower than flupentixol’s chromatographic parameters of lipophilicity. Fluphenazine’s chromatographic parameter of lipophilicity is higher than flupentixol’s chromatographic parameter of lipophilicity in two cases. The first one is fluphenazine’s chromatographic parameter of lipophilicity determined on the TLC plate RP-2F_254_ and acetone–TRIS buffer as the mobile phase which is insignificantly higher than flupentixol’s chromatographic parameter of lipophilicity determined in the same conditions. The second one is fluphenazine’s chromatographic parameter of lipophilicity determined on the TLC plate RP-18F_254_ and acetonitrile–TRIS buffer as the mobile phase which is higher than flupentixol’s chromatographic parameter of lipophilicity determined in the same conditions. This confirms the influence of chemical structure on the lipophilicity of the studied compounds and may be useful in the preliminary design of new derivatives of the investigated neuroleptics, such as the one presented in Figure 2. 

**Figure 6:**
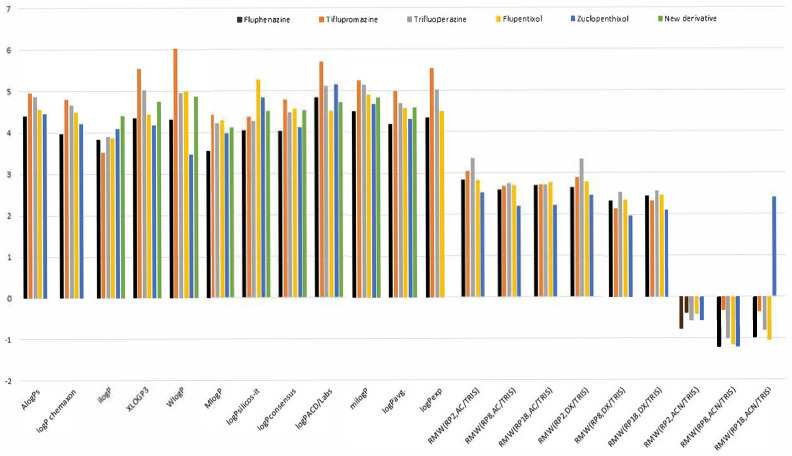
Comparison of all lipophilicity parameters obtained by means of both theoretical and chromatographic methods: logP values predicted by means of different algorithms and R_MW_ values determined using acetone–TRIS buffer (AC/TRIS), acetonitrile–TRIS buffer (ACN/TRIS) and 1,4-dioxane–TRIS buffer (DX/TRIS) as mobile phases on RP-2F_254_, RP-8F_254_ and RP-18F_254_ plates.

To fully compare the lipophilic properties of the tested compounds and the new derivative, we presented all the obtained lipophilicity parameters (logP and R_MW_ values) in the form of a graph in Figure 6.

Further analysis of the chromatographic parameters listed in Figure 6 indicates that the most optimal phases among those tested are acetone–TRIS buffer and 1,4-dioxane–TRIS buffer, which enable the attainment of reliable values of this parameter on all chromatographic plates tested (RP-2F_254_, RP-8F_254_, RP-18F_254_). These mobile phases were also successfully applied in a previous lipophilicity study of newly synthesized phenothiazine derivatives [17]. However, the mobile phase composed of acetonitrile and TRIS buffer was less useful for studying lipophilicity. Using this method, as shown in Figure 6, R_MW_ values were obtained that differed completely from the other experimental and theoretical logP values. These conclusions will be important for the further planned lipophilicity experiments of a series of newly designed molecules, such as the new one containing quinoline and a phenothiazine system presented in Figure 2. When considering all stationary phases, the lipophilicity values, notably the R_MW_ values, for RP-8F_254_ plates are comparable to those for the RP-18F_254_ plates determined by means of acetone–TRIS buffer and 1,4-dioxane–TRIS buffer, while the values obtained from RP2-F_254_ plates are only slightly higher for some neuroleptics, such as trifluoroperazine. This fact confirms in particular the influence of the mobile phase used on the assessment of the lipophilicity of the studied neuroleptics.

Furthermore, to compare the relationships between logP values and chromatographic lipophilicity parameter R_MW_ values for the tested compounds, a correlation matrix of all chromatographic lipophilicity parameters was performed. The results of the correlation matrix are presented in Table S7 (Supplementary Materials).

Analysis of the data listed in Table S7 shows that among all R_MW_ values, the most significant linear correlations with theoretical values of logP in the form of logP_silicos-it_ are shown for the chromatographic parameter determined on RP-18F_254_ plates developed by using the mobile phase consisting of acetone and TRIS buffer (RMW(RP18,AC/TRIS)—R = 0.9998. The next satisfactory correlation with both, i.e., MlogP and logP_Consensus_, is shown for R_MW_ obtained on RP-2F_254_ plates using acetonitrile and TRIS buffer, i.e., RMW (RP2, ACN/TRIS). In this case, R is 0.9636 and 0.9697, respectively. High correlation with XlogP3 (R = 0.9623) and logP_ACD/Labs_ (R = 0.9769), as well as with logP_exp_ (R = 0.9564), indicates RMW (RP18, ACN/TRIS).

Good correlations can be also observed between certain R_MW_ values like, for example, RMW (RP2, DX/TRIS) and RMW (RP2, AC/TRIS) with R = 0.9724. Excellent correlations with R = 0.9999 were seen for both RMW (RP18, DX/TRIS) and RMW (RP8, DX/TRIS). However, R_MW_ obtained by means of acetonitrile and TRIS buffer on RP18F_254_ plates, i.e., RMW (RP18, ACN/TRIS), shows a statistically significant correlation with R_MW_ determined by using the same mobile phase and chromatographic plate RP-8F_254_ (R = 0.9809). 

These results justify the use of various chromatographic plates and mobile phases to find the optimal conditions for the lipophilicity study of examined neuroleptics and their derivatives in the future.

Based on previous work [29], in the next stage, we attempted to correlate the previously calculated topological indices with the theoretical and chromatographic lipophilicity parameters of the compounds under study. The best linear correlations between partition coefficients (logP) and R_MW_ values with topological indices for the analyzed compounds are shown in Table S8.

Analysis of the data listed in Table S8 confirms that the logP values predicted by logP_ACD/Labs_ have the highest correlation (Equation (S4)) with the topological index (M), with R^2^ = 98.00 as follows: logP_ACD/Labs_ = 11.079 − 0.027∙M. A high correlation with R^2^ in the range of 84.23 to 93.81% was also found between logP_ACD/Labs_ and other topological indices calculated for studied compounds such as ^0^*χ*, ^1^*χ*, ^0^*χ*^ν^, W, R and A (Equations (S5)–(S10)). Equations (S1)–(S3), as well as Equations (S11) and (S12), show that other theoretically obtained partition coefficients such as XLOGP3, WlogP, logP_consensus_ and logP_avg_ correlate well with the Pyka indices ^0^B and ^1^B (R^2^ = 81.22–96.24). Interestingly, the topological indices newly calculated in this work, especially the Gutman index M^ν^, indicate a significant correlation with the chromatographic, i.e., experimental, lipophilicity parameter (R_MW_) obtained for RP-18 (AC/TRIS) and RP-18 (DX/TRIS) systems which consisted of RP-18F_254_ plates and acetone-AC and 1,4-dioxane-DX as organic modifiers (Equations (S13) and (S14)). Thanks to these linear models, the experimental values of zuclopentixol and a new derivative may be determined without experiment. 

To confirm this fact, we predicted the chromatographic parameters R_MW_ for both compounds using the obtained linear models, i.e., Equations (S13) and (S14), and compared them with the chromatographic values. The R_MW (RP-18 (AC/TRIS))_ predicted using Equation (S13) for zuclopenthixol is 2.229; the experimental value obtained in this work is 2.218 (Table S8). The predicted R_MW (RP-18 (DX/TRIS))_ using Equations (S14) is 1.978, and the experimental value is 2.110 (Table S8). In the case of the new derivative, the chromatographic parameters calculated based on Equations (S13) and (S14) are 2.841 and 2.386. Good agreement can also be observed between the logP values calculated by means of other equations, as shown in Table S8, for example, Equation (S1). The XlogP3 calculated using Equation (S1) for zuclopentixol is 4.45, and the one obtained from SwissADME is 4.18 [25]. Similarly, for the new derivative XlogP3 calculated according to Equation (S1), it is 4.34, and from SwissADME, it is 4.76 [25].

The newly calculated topological indices were also satisfactorily related to their selected physicochemical parameters such as molar mass, molar refractivity, molar volume and polarizability, with R^2^ placed in the range of 84.35–99.19% Equations (S15)–(S32). The best correlation was observed between index W and R with molar mass Equations (S19) and (S20), Table S9.

In further steps, we correlated the topological indices with other ADMET parameters from the Percepta platform, such as logKp, logBB, logPS and Caco-2 [28]. The correlation equations are presented in Table S10 (Equations (S33)–(S38)). As can be observed, the highest correlation is between the topological index ^1^B and logKp values (R^2^ = 95.15) and Caco-2 (R^2^ = 94.57). Other topological indices which correlated well with these parameters are the indices M, A and ^1^χ^ν^. This situation confirms the utility of the obtained linear models for rapid prediction of both ADMET parameters for the members of the studied groups of neuroleptic agents or their new derivatives.

**Figure 7:**
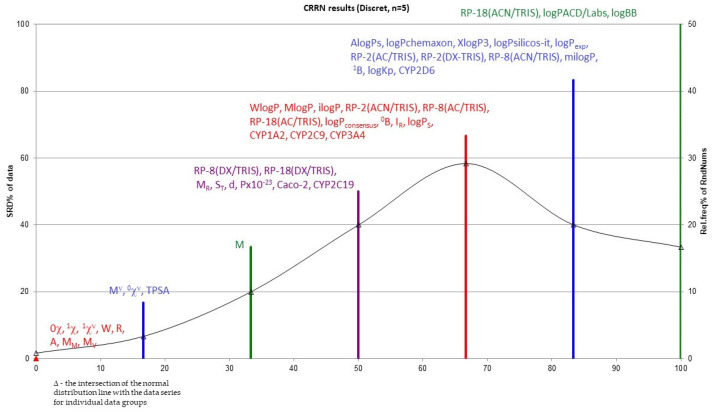
SRD analysis of values of data describing the studied compounds.

We also used one of the non-parametric analysis methods to analyze the data obtained. It was SRD analysis, i.e., the sum of ranking differences. The study was conducted to compare data describing the group of tested drugs. The analysis proposed by Prof. Heberger and described in his works [30,31,32] provides many possibilities for analyzing the obtained results. It has already been successfully used by many researchers, for example, to study retention indices of polycyclic aromatic hydrocarbons [33], anticancer acetylene quinoline derivatives [34] or triazine derivatives [35]. In our work, we proposed SRD analysis for the evaluation of all obtained data, i.e., lipophilicity values, physicochemical properties, ADMET parameters and topological index values [36,37]. The proper graph is presented in Figure 7.

The Wiener (W), Rouvray (R) and Pyka A indices will be the best in this role. In the same group, there is also molar mass and molar volume, which are very closely related to the structure of the compound. For this entire group of parameters, SRD = 0. Other useful indices, not far from the reference for which SRD = 2, are the other topological indices, namely the Gutman valence index (M^ν^), the Randić zero-order valence index (^0^*χ*^ν^) and also TPSA (topological polar surface area) (for them, SRD = 2), as well as the Gutman index M (for which SRD = 4). This confirms the validity of calculating structural descriptors, particularly topological indices, to describe chemical compounds, including compounds with pharmacological activity. It turns out that the most useful topological indices include both those based on the adjacency matrix (Gutman and Randić) and those based on the distance matrix—Wiener, Rouvray and Pyka A.

## 3. Materials and Methods

### 3.1. Reagents and Materials

The reference standards (purity ≥ 98%) of five investigated compounds, including fluphenazine, triflupromazine, trifluoperazine, flupentixol and zuclopenthixol in the form of hydrochlorides, respectively, were provided by Sigma-Aldrich (St. Louis, MO, USA). Working standard solutions of the cited compounds at a concentration of 2 mg/mL were prepared in methanol. Buffer TRIS (tris(hydroxymethyl)aminomethane) with a pH of 7.4 was purchased from Fluka (Buchs, St. Gallen, Switzerland). The HPLC-grade solvents distilled water, acetonitrile, acetone and 1,4-dioxane were from POCH (Gliwice, Poland). TLC plates were precoated with silica gel RP-2F_254_, silica gel RP-8F_254_ and silica gel RP-18F_254_ (Merck, Darmstadt, Germany).

### 3.2. TLC Analysis

The chromatographic study was carried out on 10 × 10 cm plates developed using the following mixtures: acetone–buffer TRIS, acetonitrile–buffer TRIS and 1,4-dioxane–buffer TRIS (*v*/*v*). The content of organic modifier varied from 40% to 90% in 5% increments. Five microliters of each sample solution was spotted as separate spots with a 5 mm width. Plates were developed at ambient temperature (20 ± 2 °C) to a distance of 70 mm in a horizontal chamber (Camag, Muttenz, Switzerland) and then visualized under UV light (λ = 254 nm). All measurements were performed in triplicate.

### 3.3. The Calculation of Chromatographic Parameter of Lipophilicity (R_MW_)

The mean value of the retardation factor (R_f_) of each compound was used to calculate the R_M_ values:

(1) $$R_{M}=log(\frac{1-R_{f}}{R_{f}})$$

Next, the R_M_ values obtained for each compound by means of a proper mobile phase containing acetone, acetonitrile and 1,4-dioxane and buffer TRIS (pH = 7.4) were extrapolated to pure water in accordance with the Soczewiński–Wachtmeister equation:

(2) $$R_{M}=R_{MW}+S\cdot \varphi$$

where S is the slope of the curve; φ is the concentration of organic modifier in the mobile phase; and R_MW_ is the R_M_ value extrapolated to pure water in the mobile phase used. The characteristic of the obtained linear plots are given in the Supplementary Materials as Tables S2–S6.

### 3.4. The Calculation of LogP Values and Other Physicochemical Parameters

In parallel with the chromatographic analyses, the theoretical values of partition coefficients expressed as logP (AlogPs, ilogP, XlogP3, WlogP, MlogP, milogP, logPsilicos-it, logPconsensus, logP_chemaxon_ and logP_ACD/Labs_) were determined using different web servers and physics-based methods, as well as atom and fragment/topological approaches available as SwissADME, Molinspiration Cheminformatics, DrugBank servers and the ChemSketch program [24,25,26,27,38]. Based on logP values obtained from ten different algorithms, the average value was also calculated and marked as logP_avg._ The source of the experimental partition coefficient values (i.e., logP in n-octanol-water) designated as logP_exp_, for all the compounds tested except zuclopenthixol and the proposed new derivative, was DrugBank [24]. Molar mass [g/mol], molar refractivity [cm^3^], molar volume [cm^3^], index of refraction, surface tension [dyne/cm], density [g/cm^3^], polarizability [cm^3^] and TPSA [Å^2^] were obtained using ChemSketch and Molinspiration Cheminformatics [26,27]. In addition to this, the Percepta program was useful to predict other ADMET parameters of the tested compounds such as logBB, logKp, logPS, Caco-2 in [cm/s] and others, CYP1A2, CYP2C9, CYP2C19, CYP3A4 and CYP2D6 inhibitors [28].

### 3.5. Topological Indices

The following topological indices based on the distance matrix and the adjacency matrix, respectively, such as Pyka (A, ^0^B, ^1^B), Wiener (W), Rouvray–Crafford (R), Gutman (M, M^ν^), Randić (^0^χ, ^1^χ, ^0^χ^ν^, ^1^χ^ν^), were calculated using the formulas presented in detail in our previous papers [29].

The Pyka indices (A, ^0^B, ^1^B) and Wiener index (W) were calculated as below:

(3) $$A=\sqrt{\sum_{l}^{n_{m}}{{\left(S_{i}\right)}_{l}}^{2}}$$

(4) B0=∑lnm(Si)l−1/2

(5) B1=∑lnm(SiSj)l−1/2

(6) $$W=\sum_{i}d_{ii}+\sum_{i<j}d_{ij}$$

where l is the number identifying the subgraphs; S_i_ and S_j_ are the sums of the distances of vertex i and vertex j from all other vertices of the graph; and i and j are the neighboring vertices in the subgraph.

The Rouvray–Crafford index (R) is stated as

(7) $$R=\sum_{i}d_{ii}+\sum_{ij}d_{ij}$$

Next, indices such as the Gutman index M and Randić’ indices ^0^χ and ^1^χ are given by

(8) $$M=\sum_{i=l}^{N}{\left(\delta_{i}\right)}^{2}$$

(9) χm=∑j=lnm∏i=lm+1(δi)j−1/2

where *δ* represents the degrees of vertices (*δ*_i_ is the i-th degree of vertices in a graph and means the number of neighboring vertices or the number of edges falling on a vertex), m takes the value 0 or 1 and *n*_m_ represents the number of connections.

The Gutman index M^ν^ and Randić indices ^0^*χ*^ν^ and ^1^*χ*^ν^ can be proved by using the following formula:

(10) $$M^{\nu }=\;\sum_{i=l}^{N}{{(\delta }_{i}^{\nu })}^{2}$$

(11) χmν=∑j=lnm∏i=lm+l(δiν)j−1/2

where *δ*^ν^ is related to the number of valence electrons of the atom that forms the vertex of the graph and can be calculated by using this formula:

(12) $$\delta^{\nu }=Z^{\nu }-h$$

The symbol *Z*^ν^ is the number of valence electrons of the atomic vertex, while h is the number of hydrogen atoms associated with the vertex. The next symbol ν shows that the degree of vertex δ was calculated on the basis of *Z*^ν^.

### 3.6. Statistical Analysis of Data

The cluster analysis (Euclidean distance) of the obtained lipophilicity parameters was performed using the Statistica program version 13.3 [39]. Additionally, we used the sum of ranking differences analysis (SRD) as a useful statistical non-parametric method for comparing data based not on raw data but on ranks [36,37]. The SRD analysis of topological indices and values of lipophilicity, as well as the physicochemical and ADME parameters of the examined compounds, was performed using a Microsoft Excel macro program downloaded at http://aki.ttk.mta.hu/srd/ (accessed on 10 January 2025).

## 4. Conclusions

In this work, the importance of the hybrid method, i.e., chromatographic methods such as RP-TLC and calculation for evaluation of the lipophilicity and other ADMET properties of the neuroleptics fluphenazine, triflupromazine, trifluoperazine, flupentixol and zuclopenthixol, was discussed. We employed the chromatographic parameters (R_MW_) using different stationary and mobile phases. Analysis of the obtained R_MW_ values indicates that the most optimal phases among those tested are acetone–TRIS buffer and 1,4-dioxane–TRIS buffer, which enable the attainment of reliable values of this parameter on all chromatographic plates tested (RP-2F_254_, RP-8F_254_, RP-18F_254_). The difference between the calculated logP values obtained using different platforms and algorithms such as AlogPs, ilogP, XlogP3, WlogP, MlogP, milogP, logPsilicos-it, logP_consensus_, logP_chemaxon_ and logP_ACD/Labs_ confirms that the theoretical methods are attractive from the economic point of view but require comparison with experimental ones. Additionally, the newly calculated topological indices for the studied molecules were also related to experimental and theoretical chromatographic parameters of lipophilicity, as well as other ADMET parameters of these compounds. Strong correlation between the selected topological indices and R_MW_, as well as logBB and logKp, is significant and valuable for designing future derivatives like those proposed that consisted of a quinoline ring. Linear models obtained based on Gutman’s index (M^ν^) enable quick and accurate prediction of experimental values of lipophilicity parameters for the studied group of pharmaceuticals.

## Figures and Tables

**Table 1:** The physicochemical parameters defined by using ChemSketch and Molinspiration Cheminformatics [26,27].

Compound	Molar Mass [g/mol]	Molar Refractivity [cm^3^]	Molar Volume [cm^3^]	Index of Refraction	Surface Tension [dyne/cm]	Density [g/cm^3^]	Polarizability [cm^3^]	Topological Polar Surface Area TPSA [Å^2^]
Fluphenazine	437.52	114.30	343.8	1.579	44.0	1.272	4.53∙10^−23^	31.64
Triflupromazine	352.42	92.83	284.4	1.566	39.1	1.238	3.68∙10^−23^	8.17
Trifluoperazine	407.50	108.15	328.7	1.571	40.3	1.239	4.29∙10^−23^	11.41
Flupentixol	434.52	116.90	332.4	1.620	49.8	1.306	4.63∙10^−23^	26.70
Zuclopenthixol	400.96	116.80	310.8	1.675	58.1	1.289	4.63∙10^−23^	26.70
New derivative	488.57	130.23	371.2	1.619	49.8	1.316	5.16∙10^−23^	44.53

**Table 2:** The lipophilicity parameters calculated using the online databases: DrugBank, SwissADME, ChemSketch and Molinspiration Cheminformatics [24,25,26,27].

Compound	AlogPs [24]	logP Chemaxon [24]	ilogP [25]	XLOGP3 [25]	WlogP [25]	MlogP [25]	logPsilicos-it [25]	logPconsensus [25]	logPACD/Labs [26]	milogP [27]	logPa_vg_	logP_exp_ [24]
Fluphenazine	4.40	3.97	3.83	4.36	4.32	3.57	4.05	4.03	4.84	4.51	4.20 ± 0.35	4.36
Triflupromazine	4.95	4.81	3.52	5.54	6.03	4.43	4.37	4.78	5.70	5.25	4.99 ± 0.72	5.54
Trifluoperazine	4.87	4.66	3.91	5.03	4.96	4.21	4.27	4.47	5.11	5.14	4.70 ± 0.42	5.03
Flupentixol	4.56	4.50	3.88	4.44	4.99	4.28	5.27	4.57	4.51	4.91	4.58 ± 0.37	4.51
Zuclopenthixol	4.46	4.22	4.10	4.18	3.47	3.97	4.84	4.11	5.14	4.69	4.32 ± 0.48	-
New derivative	-	-	4.41	4.76	4.87	4.11	4.51	4.53	4.71	4.83	4.59 ± 0.25	-

**Table 3:** The ADME parameters obtained by using the Percepta platform [28].

Compound	logBB	logKp	logPS	Caco-2 [cm/s]	CYP1A2 Inhibitor	CYP2C9 Inhibitor	CYP2C19 Inhibitor	CYP2D6 Inhibitor	CYP3A4 Inhibitor
Fluphenazine	0.565	−2.744	−1.934	209∙10^−6^	0.76	0.08	0.34	0.84	0.44
Triflupromazine	0.792	−2.557	−1.371	53∙10^−6^	0.84	0.09	0.15	0.85	0.50
Trifluoperazine	0.847	−2.730	−1.541	204∙10^−6^	0.76	0.09	0.68	0.85	0.65
Flupentixol	0.564	−2.753	−1.190	212∙10^−6^	0.70	0.13	0.41	0.67	0.59
Zuclopenthixol	0.612	−2.755	−1.642	221∙10^−6^	0.54	0.15	0.38	0.68	0.54
New derivative	0.106	−2.737	−1.940	197∙10^−6^	0.43	0.12	0.33	0.72	0.53

**Table 4:** The topological indices of the tested compounds.

Compound	Topological Indices Based on Adjacency Matrix						Topological Indices Based on Distance Matrix				
	M	M^ν^	^0^χ	^1^χ	^0^χ^ν^	^1^χ^ν^	W	R	A	^0^B	^1^B
Fluphenazine	230	478	19.8450	12.6645	16.7367	10.6957	2162.203	4322.352	814.1559	2.5506	0.2492
Triflupromazine	198	406	16.0250	9.9854	14.4997	7.7646	993.109	1984.308	418.5506	2.7009	0.3498
Trifluoperazine	222	446	18.4308	11.6266	15.8753	10.1492	1679.611	3357.168	653.6544	2.6108	0.2768
Flupentixol	242	474	19.7279	12.3758	18.1718	10.2009	2016.396	4030.632	758.0177	2.6407	0.2668
Zuclopenthixol	224	324	17.1379	11.1258	15.4038	9.5729	1619.394	3236.980	640.4593	2.5103	0.2604
New derivative	264	528	21.9998	14.0693	18.0542	11.5420	2802.912	5603.378	993.7961	2.7087	0.2518

**Table 5:** The chromatographic parameters of lipophilicity (R_MW_) determined by the use of RP-TLC on TLC plates RP-2F_254_, RP-8F_254_ and RP-18F_254_ as the stationary phase and acetone–TRIS buffer, acetonitrile–TRIS buffer and 1,4-dioxane–TRIS buffer as the mobile phase.

Mobile Phase	Chromatographic Plates		
	RP-2F_254_	RP-8F_254_	RP-18F_254_
**Fluphenazine**			
Acetone–TRIS buffer	2.834	2.591	2.702
Acetonitrile–TRIS buffer	−0.777	−1.230	−0.995
1,4-dioxane–TRIS buffer	2.653	2.340	2.454
**Triflupromazine**			
Acetone–TRIS buffer	3.046	2.690	2.718
Acetonitrile–TRIS buffer	−0.405	−0.334	−0.361
1,4-dioxane–TRIS buffer	2.890	2.153	2.342
**Trifluoperazine**			
Acetone–TRIS buffer	3.348	2.754	2.714
Acetonitrile–TRIS buffer	−0.568	−1.023	−0.810
1,4-dioxane–TRIS buffer	3.333	2.547	2.580
**Flupentixol**			
Acetone–TRIS buffer	2.829	2.702	2.762
Acetonitrile–TRIS buffer	−0.427	−1.165	−1.058
1,4-dioxane–TRIS buffer	2.782	2.363	2.468
**Zuclopenthixol**			
Acetone–TRIS buffer	2.529	2.206	2.218
Acetonitrile–TRIS buffer	−0.582	−1.206	2.411
1,4-dioxane–TRIS buffer	2.459	1.981	2.110

## Data Availability

The data are available from the corresponding authors upon reasonable request.

## Supplementary Materials

The following supporting information can be downloaded at: https://www.mdpi.com/article/10.3390/ph18091255/s1, The supplementary materials are available online. Table S1. Correlation matrix of logP values obtained by using different methods [24,25,26,27]. The relationships in bold are statistically significant (*p* < 0.0500); Table S2. Data for linear equation between content of the organic modifier and R_M_ values for zuclopenthixol obtained by using the Soczewiński–Wachtmeister equation R_M_ = R_MW_ + S∙φ, where SE—standard error, R^2^—coefficient of determination, SEE—standard error of estimate, Fisher F distribution value, *p*—significance level, φ—volume fraction of the organic modifier in the mobile phase; Table S3. Data for linear equation between content of the organic modifier and R_M_ values for flupentixol obtained by using the Soczewiński–Wachtmeister equation R_M_ = R_MW_ + S∙φ, where SE—standard error, R^2^—coefficient of determination, SEE—standard error of estimate, Fisher F distribution value, *p*—significance level, φ—volume fraction of the organic modifier in the mobile phase; Table S4. Data for linear equation between content of the organic modifier and R_M_ values for triflupromazine obtained by using the Soczewiński–Wachtmeister equation R_M_ = R_MW_ + S∙φ, where SE—standard error, R^2^—coefficient of determination, SEE—standard error of estimate, Fisher F distribution value, *p*—significance level, φ—volume fraction of the organic modifier in the mobile phase; Table S5. Data for linear equation between content of the organic modifier and R_M_ values for trifluoperazine obtained by using the Soczewiński–Wachtmeister equation R_M_ = R_MW_ + S∙φ, where SE—standard error, R^2^—coefficient of determination, SEE—standard error of estimate, Fisher F distribution value, *p*—significance level, φ—volume fraction of the organic modifier in the mobile phase; Table S6. Data for linear equation between content of the organic modifier and R_M_ values for fluphenazine obtained by using the Soczewiński–Wachtmeister equation R_M_ = R_MW_ + S∙φ, where SE—standard error, R^2^—coefficient of determination, SEE—standard error of estimate, Fisher F distribution value, *p*—significance level, φ—volume fraction of the organic modifier in the mobile phase; Table S7. Correlation matrix of chromatographic lipophilicity parameters (R_MW_) determined for all studied compounds with logP values. The results in bold are statistically significant (*p* < 0.0500). Acetone–TRIS buffer (AC/TRIS), acetonitrile–TRIS buffer (ACN/TRIS) and 1,4-dioxane–TRIS buffer (DX/TRIS) as mobile phases Table S8. Linear correlations between lipophilicity parameters and topological indices of the studied compounds. Acetone–TRIS buffer (AC/TRIS), acetonitrile–TRIS buffer (ACN/TRIS) and 1,4-dioxane–TRIS buffer (DX/TRIS) as mobile phases, R^2^—coefficient of determination, SEE—standard error of estimate, Fisher F distribution value, *p*—significance level, n—number of points; Table S9. Linear correlations between physicochemical parameters and topological indices of the studied compounds, where M_M_—molar mass, M_R_—molar refractivity, M_V_—molar volume, P—polarizability, R^2^—coefficient of determination, SEE—standard error of estimate, Fisher F distribution value, *p*—significance level, n—number of points; Table S10. Linear correlations between ADMET parameters and topological indexes of the tested compounds. R^2^—coefficient of determination, SEE—standard error of estimate, Fisher F distribution value, *p*—significance level, n—number of points.
