# Supplementary material for: Use of TLC and Computational Methods to Determine Lipophilicity Parameters of Selected Neuroleptics: Comparison of Experimental and Theoretical Studies
Source: Pharmaceuticals (Basel). 2025 Aug 24;18(9):1255. doi: 10.3390/ph18091255 (PMC12472637; doi:10.3390/ph18091255)
Supplement: Supplementary file 1 [file pharmaceuticals-18-01255-s001.zip › pharmaceuticals-3804132-supplementary.pdf]

Table. S1. Correlation matrix of logP values obtained using different methods [24-27]. The relationships in bold are statistically significant (p<0.0500).

**Partition coefficient (logP)**	**AlogPs**	**logPchemaxon**	**ilogP**	**XLOGP3**	**WlogP**	**MlogP**	**logPsilicos-it**	**logPconsensus**	**logPACD/Labs**	**milogP**	**logPa$_{vg}$**	**logP$_{exp}$**
**AlogPs**	1.0000	0.9272	-0.5146	0.9493	0.8329	0.7989	-0.1070	0.7964	0.7954	**0.9642**	0.9354	**0.9552**
**logP chemaxon**		1.0000	-0.4359	0.8273	0.8724	**0.9651**	0.2728	0.9481	0.5819	**0.9931**	**0.9741**	0.8539
**ilogP**			1.0000	-0.7428	-0.8111	-0.3931	0.2024	-0.5427	-0.8302	-0.4497	-0.6277	-0.74210
**XLOGP3**				1.0000	0.8889	0.6896	-0.2545	0.7411	0.9376	0.8722	0.9064	**0.9984**
**WlogP**					1.0000	0.8528	0.1549	0.9239	0.7650	0.8639	**0.9566**	0.9103
**MlogP**						1.0000	0.5067	**0.9844**	0.4116	0.9276	0.9298	0.7284
**logPsilicos-it**							1.0000	0.4606	-0.5176	0.1605	0.1738	-0.1990
**logPconsensus**								1.0000	0.5018	0.9113	**0.9524**	0.7782
**logPACD/ Labs**									1.0000	0.6480	0.7178	0.9202
**milogP**										1.0000	**0.9731**	0.8928
**logPa$_{vg}$**												0.9286
**logP$_{exp}$**												1.0000

Table S2. Data for linear equation between content of the organic modifier and R_M values for zuclopenthixol obtained by using the Soczewiński-Wachtmeister equation: $R_M = R_{MW} + S\cdot\varphi$, where: SE – standard error, R^2 – coefficient of determination, SEE – standard error of estimate, Fisher F distribution value, p – significance level, φ – volume fraction of the organic modifier in the mobile phase.

**Acetone-TRIS buffer (v/v)**							
**Stationary phase**	**R_{MW}±SE**	**S±SE**	**R^2 [%]**	**SEE**	**F**	**p**	**φ**
RP-2 F_{254}	2.529±0.252	-4.008±0.366	93.74	0.176	119.71	0.0000	0.40 – 0.90
RP-8 F_{254}	2.206±0.207	-3.334±0.301	93.87	0.145	122.46	0.0000	0.40 – 0.90
RP-18 F_{254}	2.218±0.238	-3.195±0.346	91.43	0.166	85.35	0.0000	0.40 – 0.90
**Acetonitrile-TRIS buffer (v/v)**							
**Stationary phase**	**R_{MW}±SE**	**S±SE**	**R^2 [%]**	**SEE**	**F**	**p**	**φ**
RP-2 F_{254}	0.582±0.089	-1.240±0.153	94.27	0.032	65.77	0.0013	0.45 – 0.70
RP-8 F_{254}	-1.206±0.139	1.315±0.240	88.26	0.050	30.08	0.0054	0.45 – 0.70
RP-18 F_{254}	2.411±0.454	-5.135±0.899	91.58	0.142	32.62	0.0106	0.40 – 0.60
**1,4-dioxane-TRIS buffer (v/v)**							
**Stationary phase**	**R_{MW}±SE**	**S±SE**	**R^2 [%]**	**SEE**	**F**	**p**	**φ**
RP-2 F_{254}	2.459±0.130	-4.185±0.202	98.85	0.070	428.30	0.0000	0.40 – 0.80
RP-8 F_{254}	1.981±0.189	-3.427±0.295	96.44	0.102	135.36	0.0001	0.40 – 0.80
RP-18 F_{254}	2.110±0.176	-3.573±0.275	97.13	0.095	169.02	0.0000	0.40 – 0.80

Table S3. Data for linear equation between content of the organic modifier and R_M values for flupentixol obtained by using the Soczewiński-Wachtmeister equation: $R_M = R_{MW} + S \cdot \varphi$, where: SE – standard error, R^2 – coefficient of determination, SEE – standard error of estimate, Fisher F distribution value, p – significance level, φ – volume fraction of the organic modifier in the mobile phase.

Stationary phase	R_{MW}±SE	S±SE	R^2 [%]	SEE	F	p	φ
**Acetone-TRIS buffer (v/v)**							
RP-2 F_{254}	2.829±0.194	-4.310±0.282	96.68	0.136	232.84	0.0000	0.40 – 0.90
RP-8 F_{254}	2.702±0.228	-3.996±0.331	94.79	0.159	145.60	0.0000	0.40 – 0.90
RP-18 F_{254}	2.762±0.175	-3.951±0.254	96.80	0.122	241.65	0.0000	0.40 – 0.90
**Acetonitrile-TRIS buffer (v/v)**							
**Stationary phase**	**R_{MW}±SE**	**S±SE**	**R^2 [%]**	**SEE**	**F**	**p**	**φ**
RP-2 F_{254}	-0.427±0.153	-1.486±0.263	88.85	0.055	31.87	0.0048	0.45 – 0.70
RP-8 F_{254}	-1.165±0.108	1.114±0.186	89.98	0.039	35.90	0.0039	0.45 – 0.70
RP-18 F_{254}	-1.058±0.077	0.788±0.128	92.65	0.020	37.82	0.0086	0.50 – 0.70
**1,4-dioxane-TRIS buffer (v/v)**							
**Stationary phase**	**R_{MW}±SE**	**S±SE**	**R^2 [%]**	**SEE**	**F**	**p**	**φ**
RP-2 F_{254}	2.782±0.094	-4.533±0.146	99.48	0.050	963.98	0.0000	0.40 – 0.80
RP-8 F_{254}	2.363±0.214	-3.888±0.333	96.46	0.115	136.05	0.0001	0.40 – 0.80
RP-18 F_{254}	2.468±0.246	-4.012±0.383	95.65	0.132	109.91	0.0001	0.40 – 0.80

Table S4. Data for linear equation between content of the organic modifier and R_M values for triflupromazine obtained by using the Soczewiński-Wachtmeister equation: $R_M = R_{MW} + S \cdot \varphi$, where: SE – standard error, R^2 – coefficient of determination, SEE – standard error of estimate, Fisher F distribution value, p – significance level, φ – volume fraction of the organic modifier in the mobile phase.

Stationary phase	R_{MW}±SE	S±SE	R^2 [%]	SEE	F	p	φ
**Acetone-TRIS buffer (v/v)**							
RP-2 F_{254}	3.046±0.271	-4.538±0.394	94.30	0.190	132.37	0.0000	0.40 – 0.90
RP-8 F_{254}	2.690±0.232	-4.053±0.338	94.73	0.162	143.82	0.0000	0.40 – 0.90
RP-18 F_{254}	2.718±0.168	-3.966±0.244	97.05	0.117	263.17	0.0000	0.40 – 0.90
**Acetonitrile-TRIS buffer (v/v)**							
**Stationary phase**	**R_{MW}±SE**	**S±SE**	**R^2 [%]**	**SEE**	**F**	**p**	**φ**
RP-2 F_{254}	-0.405±0.166	-1.306±0.286	83.95	0.060	20.92	0.0102	0.45 – 0.70
RP-8 F_{254}	-0.334±0.019	-1.018±0.036	99.75	0.004	813.69	0.0012	0.45 – 0.60
RP-18 F_{254}	-0.361±0.084	-0.931±0.140	93.68	0.022	44.44	0.0069	0.50 – 0.70
**1,4-dioxane-TRIS buffer (v/v)**							
**Stationary phase**	**R_{MW}±SE**	**S±SE**	**R^2 [%]**	**SEE**	**F**	**p**	**φ**
RP-2 F_{254}	2.890±0.232	-4.438±0.362	96.79	0.125	150.59	0.0001	0.40 – 0.80
RP-8 F_{254}	2.153±0.213	-3.654±0.331	96.06	0.114	121.77	0.0001	0.40 – 0.80
RP-18 F_{254}	2.342±0.261	-3.896±0.407	94.83	0.140	91.76	0.0002	0.40 – 0.80

Table S5. Data for linear equation between content of the organic modifier and R_M values for trifluoperazine obtained by using the Soczewiński-Wachtmeister equation: $R_M = R_{MW} + S\cdot\varphi$, where: SE – standard error, R^2 – coefficient of determination, SEE – standard error of estimate, Fisher F distribution value, p – significance level, φ – volume fraction of the organic modifier in the mobile phase.

Acetone-TRIS buffer (v/v)							
**Stationary phase**	**R_{MW}±SE**	**S±SE**	**R^2 [%]**	**SEE**	**F**	**p**	**φ**
RP-2 F_{254}	3.348±0.250	-4.793±0.364	95.60	0.175	173.66	0.0000	0.40 – 0.90
RP-8 F_{254}	2.754±0.209	-4.049±0.304	95.69	0.146	177.43	0.0000	0.40 – 0.90
RP-18 F_{254}	2.714±0.200	-3.784±0.292	95.46	0.140	168.23	0.0000	0.40 – 0.90
**Acetonitrile-TRIS buffer (v/v)**							
**Stationary phase**	**R_{MW}±SE**	**S±SE**	**R^2 [%]**	**SEE**	**F**	**p**	**φ**
RP-2 F_{254}	-0.568±0.088	-0.790±0.152	87.11	0.032	27.02	0.0065	0.45 – 0.70
RP-8 F_{254}	-1.023±0.082	0.929±0.141	91.57	0.029	43.45	0.0027	0.45 – 0.70
RP-18 F_{254}	-0.810±0.074	0.470±0.122	83.24	0.019	14.89	0.0307	0.50 – 0.70
**1,4-dioxane-TRIS buffer (v/v)**							
**Stationary phase**	**R_{MW}±SE**	**S±SE**	**R^2 [%]**	**SEE**	**F**	**p**	**φ**
RP-2 F_{254}	3.333±0.328	-4.915±0.511	94.86	0.177	92.36	0.0002	0.40 – 0.80
RP-8 F_{254}	2.547±0.277	-4.093±0.431	94.75	0.149	90.18	0.0002	0.40 – 0.80
RP-18 F_{254}	2.580±0.271	-4.128±0.422	95.04	0.146	95.82	0.0002	0.40 – 0.80

Table S6. Data for linear equation between content of the organic modifier and R_M values for fluphenazine obtained by using the Soczewiński-Wachtmeister equation: $R_M = R_{MW} + S\cdot\varphi$, where: SE – standard error, R^2 – coefficient of determination, SEE – standard error of estimate, Fisher F distribution value, p – significance level, φ – volume fraction of the organic modifier in the mobile phase.

Acetone-TRIS buffer (v/v)							
**Stationary phase**	**R_{MW}±SE**	**S±SE**	**R^2 [%]**	**SEE**	**F**	**p**	**φ**
RP-2 F_{254}	2.834±0.219	-4.355±0.319	95.88	0.154	185.94	0.0000	0.40 – 0.90
RP-8 F_{254}	2.591±0.210	-3.928±0.306	95.37	0.147	164.70	0.0000	0.40 – 0.90
RP-18 F_{254}	2.702±0.189	-3.894±0.275	96.15	0.132	199.78	0.0000	0.40 – 0.90
**Acetonitrile-TRIS buffer (v/v)**							
**Stationary phase**	**R_{MW}±SE**	**S±SE**	**R^2 [%]**	**SEE**	**F**	**p**	**φ**
RP-2 F_{254}	-0.777±0.074	-0.756±0.126	89.78	0.027	35.12	0.0041	0.45 – 0.70
RP-8 F_{254}	-1.230±0.164	1.183±0.283	81.39	0.059	17.49	0.0139	0.45 – 0.70
RP-18 F_{254}	-0.995±0.098	0.579±0.161	81.10	0.026	12.88	0.0371	0.50 – 0.70
**1,4-dioxane-TRIS buffer (v/v)**							
**Stationary phase**	**R_{MW}±SE**	**S±SE**	**R^2 [%]**	**SEE**	**F**	**p**	**φ**
RP-2 F_{254}	2.653±0.058	-4.332±0.091	99.78	0.031	2281.45	0.0000	0.40 – 0.80
RP-8 F_{254}	2.340±0.230	-3.881±0.359	95.90	0.124	116.88	0.0001	0.40 – 0.80
RP-18 F_{254}	2.454±0.279	-3.983±0.435	94.37	0.150	83.77	0.0003	0.40 – 0.80

Table S7. Correlation matrix of chromatographic lipophilicity parameters (R_{MW}) determined for all studied compounds with logP values. The results in bold are statistically significant ($p<0.0500$). Where: Acetone-TRIS buffer (AC/TRIS), acetonitrile-TRIS buffer (ACN/TRIS) and 1,4-dioxane-TRIS buffer (DX/TRIS) as mobile phases

Lipophilicity parameter	R_{MW}(RP2,AC/TRIS)	R_{MW}(RP8,AC/TRIS)	R_{MW}(RP18,AC/TRIS)	R_{MW}(RP2,DX/TRIS)	R_{MW}(RP8,DX/TRIS)	R_{MW}(RP18,DXTRIS)	R_{MW}(RP2,ACN/TRIS)	R_{MW}(RP8ACN/TRIS)	R_{MW}(RP18,ACN/TRIS)
**AlogPs**	0.7625	0.7337	-0.0943	0.7066	-0.1164	-0.1112	0.6336	0.8005	0.8288
**$logP_{Chemaxon}$**	0.592	0.8343	0.2848	0.6106	-0.1368	-0.1323	0.8661	0.7264	0.6872
**ilogP**	0.0576	0.1276	0.2093	0.2163	0.8934	0.8910	-0.4249	-0.9250	-0.8889
**XLOGP3**	0.6076	0.4866	-0.2469	0.4919	-0.3776	-0.3726	0.5560	0.9337	**0.9623**
**WlogP**	0.2771	0.4591	0.1580	0.2188	-0.5974	-0.5935	0.8297	0.9451	0.8821
**MlogP**	0.9298	0.8020	0.5162	0.4540	-0.1949	-0.1914	**0.9636**	0.6479	0.5613
**$logP_{silicos\text{-}it}$**	-0.3618	0.3541	**0.9998**	-0.1627	-0.0199	-0.0214	0.6616	-0.1543	-0.3272
**$logP_{Consensus}$**	0.3049	0.6908	0.4674	0.3365	-0.3624	-0.3589	**0.9697**	0.7506	0.6566
**$logP_{ACD/Labs}$**	0.4834	0.1741	-0.5146	0.3039	-0.4973	-0.4930	0.2886	0.9180	**0.9769**
**milogP**	0.6654	0.8272	0.1732	0.6640	-0.1125	-0.1076	0.8033	0.7484	0.7312
**$logP_{avg.}$**	0.5066	0.6906	0.1825	0.4813	-0.3378	-0.3333	0.8492	0.8617	0.8212
**$logP_{exp}$**	0.5929	0.5125	-0.1914	0.4870	-0.3856	-0.3806	0.6025	0.9378	**0.9564**
**RMW(RP2,AC/TRIS)**	1.0000	0.7407	-0.3433	**0.9724**	0.4984	0.5031	0.1333	0.2831	0.4053
**RMW(RP8,AC/TRIS)**		1.0000	0.3722	0.8467	0.4184	0.4221	0.6559	0.2466	0.2400
**RMW(RP18,AC/TRIS)**			1.0000	-0.1428	-0.0058	-0.0073	0.6666	-0.1535	-0.3249
**RMW(RP2,DX/TRIS)**				1.0000	0.6176	0.6218	0.2109	-0.1536	0.2518
**RMW(RP8,DX/TRIS)**					1.0000	**0.9999**	-0.3416	-0.6820	-0.5904
**RMW(RP18,DX/TRIS)**						1.0000	-0.3391	-0.6780	-0.5861
**RMW(RP2,ACN/TRIS)**							1.0000	0.6028	0.4733
**RMW(RP8,ACN/TRIS)**								1.0000	**0.9809**
**RMW(RP18,ACN/TRIS)**									1.0000

Table S8. Linear correlations between lipophilicity parameters and topological indices of the studied compounds. Acetone-TRIS buffer (AC/TRIS), acetonitrile-TRIS buffer (ACN/TRIS) and 1,4-dioxane-TRIS buffer (DX/TRIS) as

mobile phases, R^2 – coefficient of determination, SEE – standard error of estimate, Fisher F distribution value, p – significance level, n – number of points.

**Lipophilicity parameter**	**Topological index**	**Linear equation**	**R^2 [%]**	**SEE**	**F**	**p**	**n**	**No. eq.**
XLOGP3	^1B	$XLOGP3 = 1.124 + 12.782 \cdot {}^1B$	82.32	0.273	13.97	0.0334	5	(S1)
WlogP	^0B	$WlogP = -27.446 + 12.372 \cdot {}^0B$	96.24	0.211	76.83	0.0031	5	(S2)
$logP_{consensus}$	^0B	$logP_{consensus} = -6.111 + 4.035 \cdot {}^0B$	91.47	0.106	32.18	0.0109	5	(S3)
$logP_{ACD/Labs}$	M	$logP_{ACD/Labs} = 11.079 – 0.027 \cdot M$	98.00	0.072	147.02	0.0012	5	(S4)
$logP_{ACD/Labs}$	$^0\chi$	$logP_{ACD/Labs} = 9.503 – 0.244 \cdot {}^0\chi$	84.49	0.199	16.35	0.0272	5	(S5)
$logP_{ACD/Labs}$	$^1\chi$	$logP_{ACD/Labs} = 9.448 – 0.380 \cdot {}^1\chi$	85.43	0.193	17.59	0.0247	5	(S6)
$logP_{ACD/Labs}$	$^0\chi^v$	$logP_{ACD/Labs} = 9.973 – 0.304 \cdot {}^0\chi^v$	93.81	0.126	45.44	0.0067	5	(S7)
$logP_{ACD/Labs}$	W	$logP_{ACD/Labs} = 6.579 – 0.001 \cdot W$	85.74	0.191	18.04	0.0239	5	(S8)
$logPA_{CD/Labs}$	R	$logP_{ACD/Labs} = 6.579 – 0.0004 \cdot R$	85.74	0.191	18.04	0.0239	5	(S9)
$logP_{ACD/Labs}$	A	$logP_{ACD/Labs} = 6.803 – 0.003 \cdot A$	84.23	0.201	16.02	0.0280	5	(S10)
$logP_{avg}$	^0B	$logP_{avg} = -5.322 + 3.796 \cdot {}^0B$	82.35	0.152	13.99	0.0333	5	(S11)
$logP_{avg}$	^1B	$logP_{avg} = 2.577 + 7.061 \cdot {}^1B$	81.22	0.157	12.97	0.0367	5	(S12)
$R_{MW\ (RP\text{-}18\ (AC/TRIS))}$	M^v	$RP\text{-}18\ (AC/TRIS) = 1.257 + 0.003 \cdot M^v$	80.65	0.116	12.50	0.0385	5	(S13)
$R_{MW\ (RP\text{-}18\ (DX/TRIS))}$	M^v	$RP\text{-}18\ (DX/TRIS) = 1.330 + 0.002 \cdot M^v$	79.26	0.094	11.47	0.0429	5	(S14)

Table S9. Linear correlations between physicochemical parameters and topological indices of the studied compounds, where: M_M – molar mass, M_R – molar refractivity, M_V – molar volume, P – polarizability, R^2 – coefficient of determination, SEE – standard error of estimate, Fisher F distribution value, p – significance level, n – number of points.

Physicochemical parameter	Topological index	Linear equation	R^2 [%]	SEE	F	p	n	No. eq.
M_M	M	$M_M = -44.401 + 2.021 \cdot M$	89.99	12.528	26.97	0.0139	5	(S15)
M_M	$^0\chi$	$M_M = 44.205 + 19.875 \cdot {}^0\chi$	91.93	11.250	34.17	0.0100	5	(S16)
M_M	$^1\chi$	$M_M = 39.586 + 31.759 \cdot {}^1\chi$	97.73	5.960	129.42	0.0015	5	(S17)
M_M	$^1\chi^v$	$M_M = 126.229 + 28.972 \cdot {}^1\chi^v$	92.86	10.584	38.99	0.0083	5	(S18)
M_M	W	$M_M = 278.806 + 0.075 \cdot W$	99.19	3.557	368.73	0.0003	5	(S19)
M_M	R	$M_M = 278.870 + 0.038 \cdot R$	99.19	3.559	368.31	0.0003	5	(S20)
M_M	A	$M_M = 258.917 + 0.225 \cdot A$	98.87	4.217	261.51	0.0005	5	(S21)
M_M	^1B	$M_M = 626.008 – 781.983 \cdot {}^1B$	83.04	16.307	14.69	0.0313	5	(S22)
M_R	M	$M_R = -19.221 + 0.578 \cdot M$	84.42	4.617	16.25	0.0274	5	(S23)
M_R	^1B	$M_R = 177.831 – 242.462 \cdot {}^1B$	91.51	3.408	32.32	0.0108	5	(S24)
M_V	$^0\chi$	$M_V = 74.670 + 13.456 \cdot {}^0\chi$	92.28	7.433	35.87	0.0093	5	(S25)
M_V	$^1\chi$	$M_V = 74.000 + 21.290 \cdot {}^1\chi$	96.18	5.230	75.54	0.0032	5	(S26)
M_V	$^1\chi^v$	$M_V = 126.817 + 19.966 \cdot {}^1\chi^v$	96.57	4.955	84.49	0.0027	5	(S27)
M_V	W	$M_V = 236.046 + 0.050 \cdot W$	93.82	6.653	45.53	0.0066	5	(S28)
M_V	R	$M_V = 236.089 + 0.025 \cdot R$	93.82	6.654	45.51	0.0067	5	(S29)
M_V	A	$M_V = 222.917 + 0.148 \cdot A$	93.62	6.758	44.02	0.0070	5	(S30)
P	M	$P = -7.612 \cdot 10^{-24} + 2.291 \cdot 10^{-25} \cdot M$	84.35	0.000	16.17	0.0276	5	(S31)
P	^1B	$P = 7.050 \cdot 10^{-23} – 9.613 \cdot 10^{-23} \cdot {}^1B$	91.49	0.000	32.23	0.0108	5	(S32)

Table S10. Linear correlations between ADMET parameters and topological indexes of the tested compounds. R^2 – coefficient of determination, SEE – standard error of estimate, Fisher F distribution value, p – significance level, n – number of points.

ADME parameter	Topological index	Linear equation	R^2 [%]	SEE	F	p	n	No. eq.
logKp	M	logKp = -1.651 – 0.005·M	80.60	0.043	12.47	0.0386	5	(S33)
logKp	$^1\chi^v$	logKp = -2.049 – 0.068·$^1\chi^v$	83.83	0.039	15.55	0.0291	5	(S34)
logKp	A	logKp = -2.384 – 0.0005·A	77.57	0.046	10.37	0.0485	5	(S35)
logKp	^{1}B	logKp = -3.289 + 2.072·^{1}B	95.15	0.022	58.85	0.0046	5	(S36)
Caco-2	$^1\chi^v$	Caco-2 = -0.0004 + 0.0001·$^1\chi^v$	83.29	0.00003	14.95	0.0306	5	(S37)
Caco-2	^{1}B	Caco-2 = 0.001 – 0.002·^{1}B	94.57	0.00002	52.21	0.0055	5	(S38)
